# Early Results after Exclusion of Popliteal Aneurysms with an Endoprosthesis

**DOI:** 10.26502/fccm.92920298

**Published:** 2022-12-19

**Authors:** Elias Noory, Tanja Böhme, Ulrich Beschorner, Börries Jacques, Karlheinz Bürgelin, Christina Zürn, Thomas Zeller

**Affiliations:** 1 Kardiologie und Angiologie, Universitäts-Herzzentrum Freiburg-Bad Krozingen, Bad Krozingen, Germany

**Keywords:** Endoprosthesis, Popliteal Artery, Popliteal Aneurysm

## Abstract

**Objectives:**

To evaluate safety and efficacy of endoprosthesis implantation for the exclusion of popliteal artery aneurysm (PAA).

**Methods:**

Elective asymptomatic patients with aneurysm > 20 mm and symptomatic patients with endovascular therapy of PAA were included. The proportion of patients with critical limb ischemia (presence of rest pain or tissue loss) was high at 32.1%, 21.6% of the patients had acute ischemia with symptoms persisting shorter than 14 days. The primary study endpoint was the target lesion revascularization (TLR) rate at 12 months. Secondary endpoints included technical success, periinterventional adverse events, primary patency at 6, 12 and 24 months, TLR rate at 24 months, predictors on reintervention, change in in clinical symptoms using the Rutherford-Becker classification (RBC), amputation and mortality rate. One hundred thirty-four patients (68.3±10.6 years, 88.8% male) were treated with a Viabahn^®^ endoprosthesis (W.L. Gore & Associates Inc., Flagstaff, AZ, USA).

**Results:**

The average aneurysm diameter was 2.5±0.87 cm. In 41%, occlusion of the aneurysm was present. TLR rate was 31.3% and 38.8% after 12 and 24 months, respectively. Primary patency rates were 69.1%, 52.3% and 42.6% at 6, 12 and 24 months, respectively. Univariate logistic regression analysis revealed age as a predictor of reintervention and in the multivariable analysis it was treatment with lysis. An improvement in RBC was seen at all-time points. Two major amputations (1.5%) were performed and the mortality rate at 24 months was 5.2%.

**Conclusion:**

Primary patency rate after endovascular exclusion of PAA is low. However, limb salvage rate is high.

## 1. Introduction

The incidence of popliteal artery aneurysm (PAA) is reported to be 0.1–2.8%. It is therefore the most frequently diagnosed non-aortic aneurysm [1,2]. The Society for Vascular Surgery clinical practice guidelines on PAA 2021 recommended treatment of PAA ≥ 2 cm. [3]. Surgical and endovascular treatment options are available. A 2014 study showed that the treatment strategy for PAA varies in different countries. In Australia, 34.7% of PAAs were treated endovascularly, while in Switzerland it was 0% [4]. Small studies with less than 30 patients showed primary patency rates of up to 91% at 1 year after endovascular therapy. [5–7]. After 5 years, a primary patency rate of 82% was achieved after endovascular exclusion of a PAA using an endoprosthesis [8]. The aim of the present retrospective study is to evaluate the early results after exclusion of PAA using an endoprothesis in a large single-center cohort with a high proportion of occluded PAAs.

## 2. Material and Methods

### 2.1 Patient Population

Patients, who were treated with a Viabahn^®^ endoprosthesis (W.L. Gore & Associates Inc., Flagstaff, AZ, USA) due to PAA between January 2008 and December 2019 were included in the analysis. The local ethics committee approved the study.

### 2.2 Endovascular Technique

**Figure 1:**
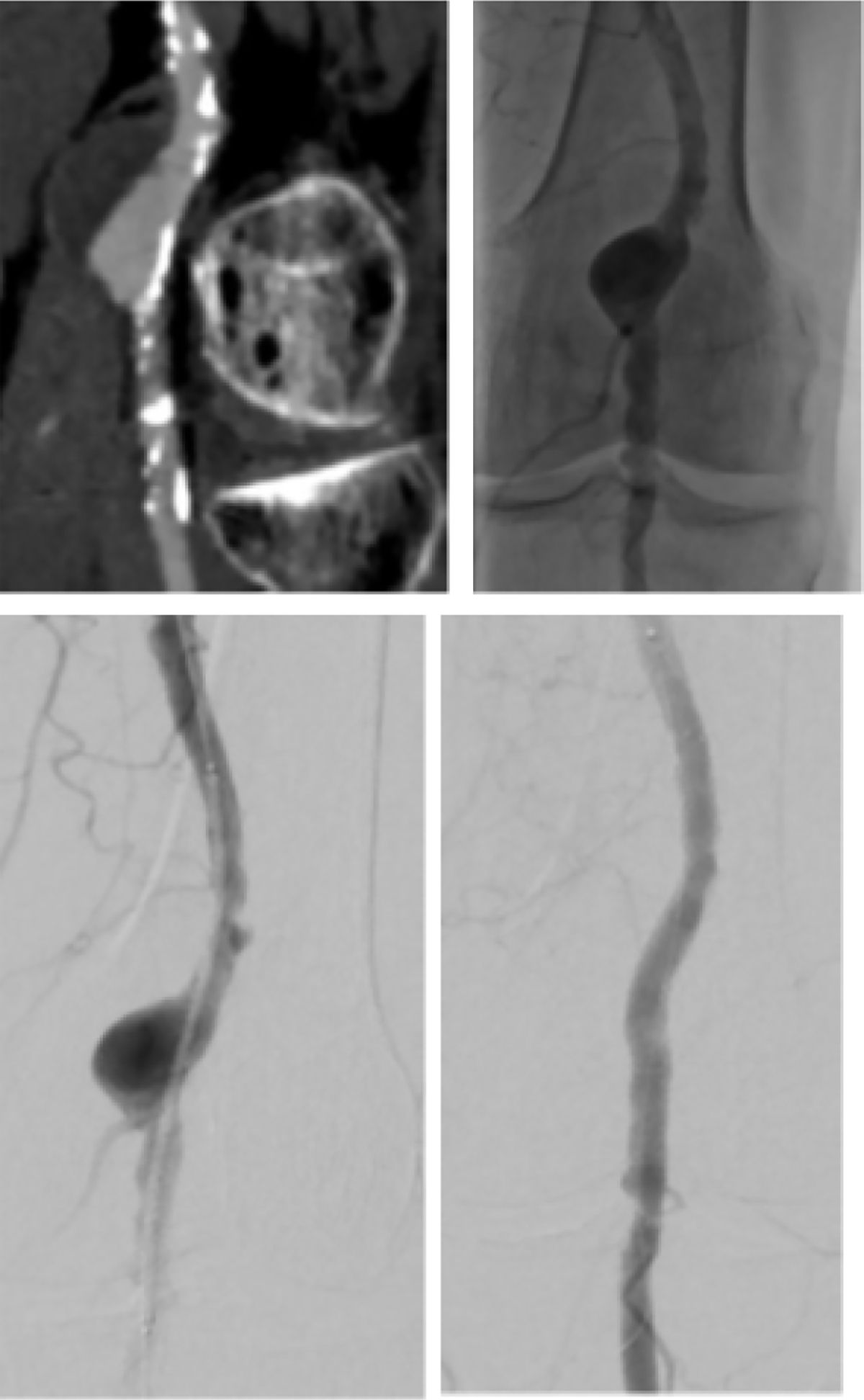
A: CTA of the left popliteal artery. The popliteal artery shows an aneurysm with partial thrombosis, B: Angiogram shows the perfused PAA, C: Positioning of the endoprosthesis, D: perfused Endoprosthesis, some retrograde perfusion is seen, this is accepted as perfusion of the aneurysm is sealed by the endoprosthesis. PAA: Popliteal artery aneurysm

Preinterventional imaging (duplex ultrasound or CT angiography) was available for all patients. Initially, an angiogram was performed to evaluate outflow vessels and to confirm the landing zones. The size of the endoprosthesis was selected according to the preprocedural imaging and angiogram. The endoprosthesis was oversized by about 15%. The length of the endoprosthesis should be chosen to enable its distal and proximal ends to be anchored in the healthy, normal-sized section of the vessel. Finally, post deployment dilatation was performed. The balloon size was chosen according to the diameter of the endoprosthesis. It was ensured that the balloon did not exceed the ends of the endoprosthesis. In the case of occluded PAA, an intraluminal occlusion passage was performedfollowed by Rotarex (BD, Franklin Lakes, NJ, USA) thrombectomy. Then the endoprosthesis could be implanted. In the case of residual thrombotic material or thrombosed below the knee vessels, a lysis therapy was performed over several hours. The decision to initiate lysis therapy was made by the physician. The Cragg-McNamara^™^ Micro Therapeutics Infusion Catheter (Medtronic, MN, USA) was used. Agent for thrombolysis was recombinant tissue plasminogen activator. After a bolus, the flow rate was determined individually. Following lysis an angiogram was performed and, if necessary, the outflow treated. In case of stenoses being present, these were treated by balloon or drug-coated balloon angioplasty. A treatment example is shown in Figure 1. After treatment, patients received 100 mg Aspirin daily for life and 75 mg/d Clopidogrel for at least 6 months. If anticoagulation was indicated (vascular or cardiac indication), it was supplemented with Clopidogreal for 6 months.

### 2.3 Study Endpoints

Primary endpoint of the study is target lesion revascularization (TLR) rate at 12 months. TLR was defined as re-intervention in the section of the treated popliteal artery. Secondary endpoints are technical success (defined as absence of flow in the PAA after endoprosthesis implantation) and periinterventional adverse events (included access site complication, perforation, distal embolization and compartment syndrome, adverse event severity assessment according to the classification system of the Society of Interventional Radiology [9]), as well as primary patency at 6,12 and 24 months (defined as a stenosis < 50%, duplex ultrasonography-derived peak systolic velocity ratio ≤2.4 without a TLR until follow-up visit), TLR rate at 24 months, predictors on the reintervention, change in clinical symptoms using the Rutherford-Becker classification (RBC) [10], change in ankle-brachial- index (ABI), amputation rate and all-cause death rate.

### 2.4 Statistical Analysis

For the analysis SPSS software (version 25.0; SPSS, Chicago, IL, USA) was used. Continuous data are presented as means ± standard deviation and compared with the t-test; categorical data are given as counts (percentages). Freedom from TLR was determined by Kaplan-Meier analysis and compared with the Mantel-Cox log-rank test. Kaplan-Meier analysis was performed for the entire cohort and for occluded PAA vs. non-occluded PAA. For investigation of predictive value of confounding variables binary logistic regression analysis was used by means of a stepwise forward variable selection procedure. In addition to patient and lesion characteristics, treatment options are included in the analysis. Results of the regression analysis are given as odds ratio with 95% confident intervals. Statistical significance was set at p<0.05.

## 3. Results

**Figure 2:**
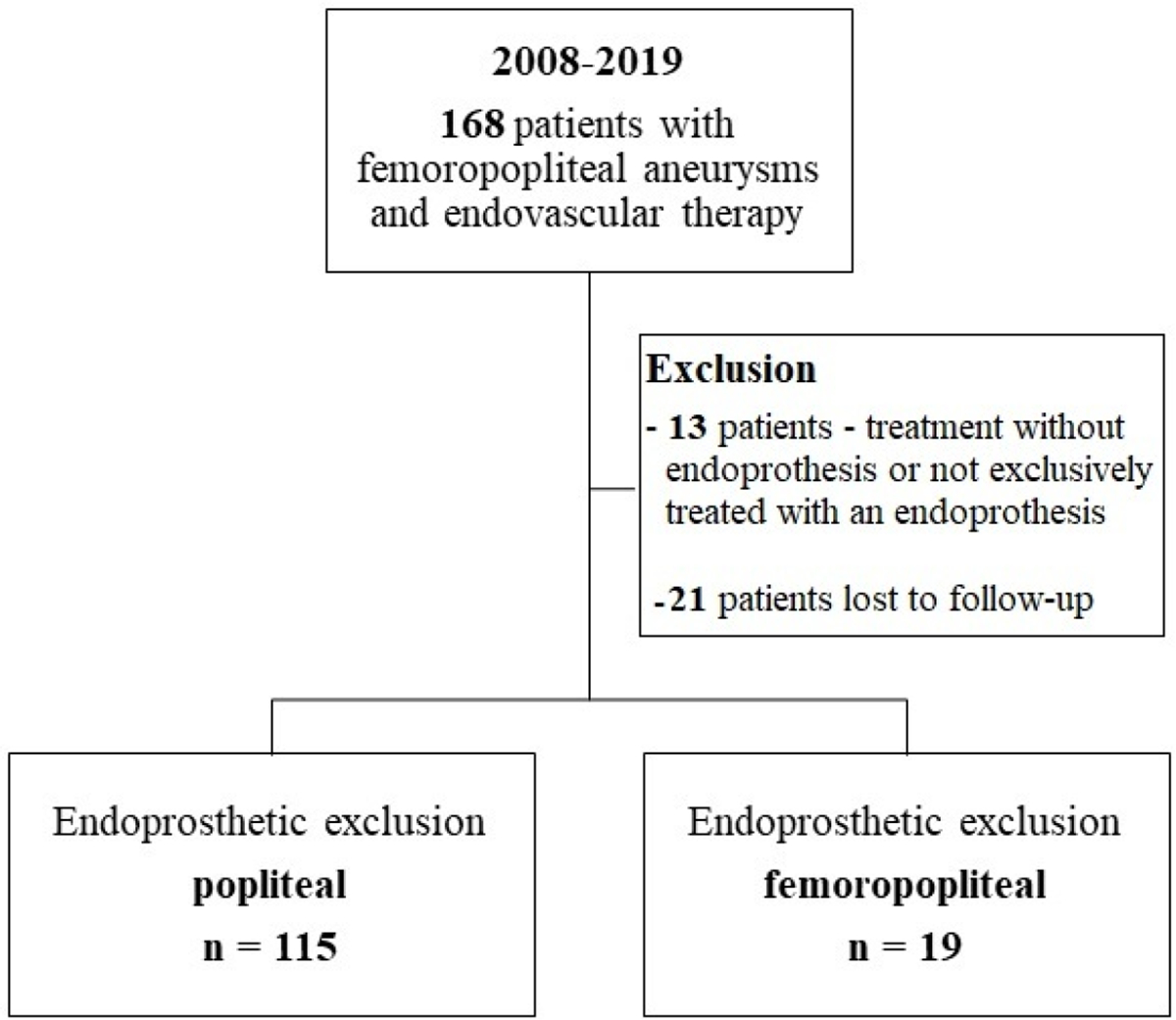
Study Flow Chart.

**Figure 3:**
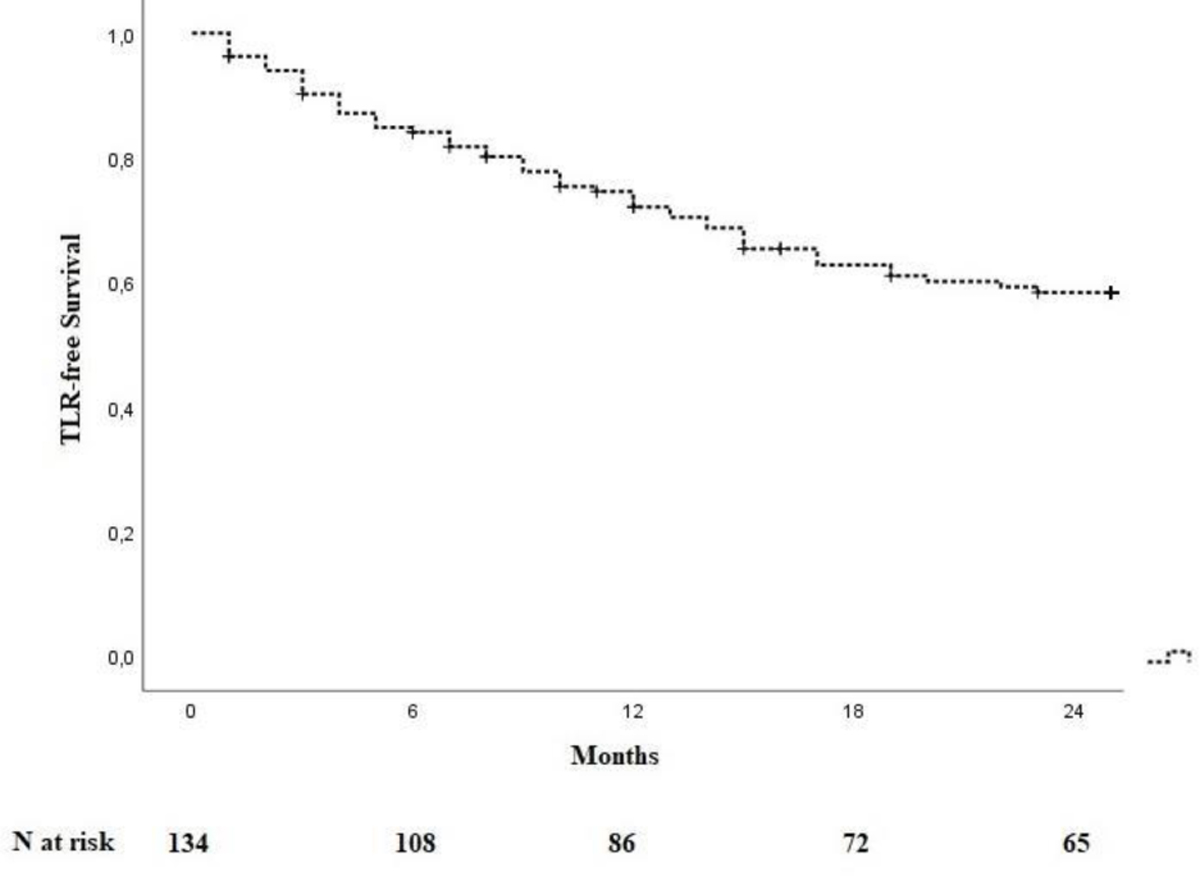
Kaplan-Meier curve for TLR-free survival. TLR – target lesion revascularization.

**Figure 4:**
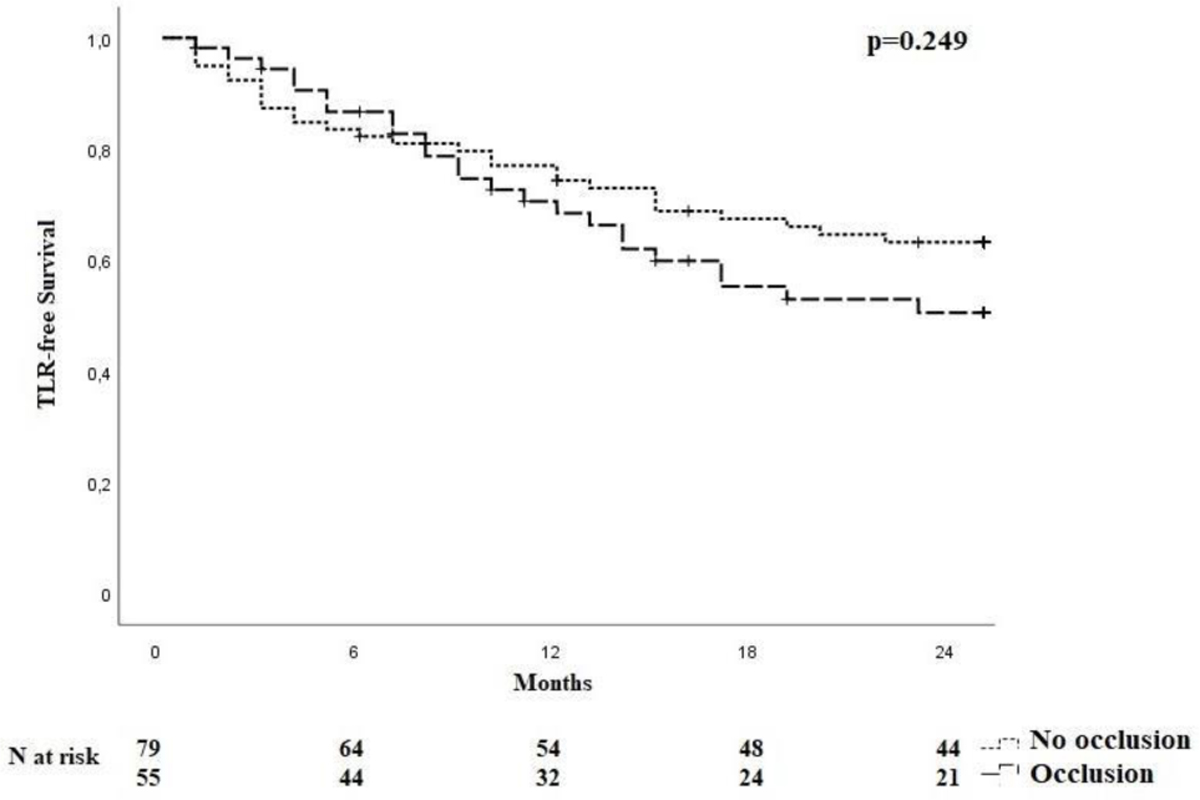
Kaplan-Meier curve for TLR-free survival for patients with an occluded PAA and non-occluded PAA. PAA: Poplitealaneurysm, TLR: Target Lesion Revascularization

One hundred and thirty-four patients were included in the analysis. The study flow chart is shown in Figure 2. Baseline characteristics are given in Table 1. The majority of patients were claudicants (61.2%). However, almost one third of the patients had critical limb ischemia (CLI) with the presence of rest pain or tissue loss (32.1%). A total of 29 patients had acute lower-extremity ischemia (ALI) with symptom onset in the last 14 days. In 55 cases the aneurysm was occluded. Therof 42.1% of patients suffered rest pain, 10.5% presented with ulcerations and 47.4% showed claudication. In most cases (68.7%), more than one segment of the popliteal artery was involved. Focal aneurysms were most frequently localized in the P2 segment of the popliteal artery (19.4%). In 88 cases (65.7%) one endoprosthesis was used to exclude the aneurysm. Two endoprostheses were implanted in 40 cases (29.9%) and three in 6 cases (4.5%). The cumulative endoprosthesis length varied from 50 to 450 mm. Lesion and index procedure characteristics are given in Table 2. In all cases, the aneurysm was completely excluded, resulting in a technical success rate of 100%. The most common complication was compartment syndrome (6.7%), followed by complications of the access site (4.5%) (Table 3). Seven complications are classified as mild, six as moderate, and nine as severe adverse events. All patients developing compartment syndrome showed an occlusion of the PAA preinterventionally, seven had ALI, and two had CLI. All patients underwent lysis and all but one underwent additional treatment of the lower leg vessels. One of the patients with compartment syndrome had TLR during follow-up. Forty-two patients required a reintervention at 12 months and 10 further patients (n=52) at 24 months. Therof, two interventions were performed due to prognostic (critically reduced foot perfusion) and the remainder due to symptomatic indication. This results in a TLR rate of 31.3% and 38.8% after 12 and 24 months, respectively (Table 4). The TLR-free survival estimated by Kaplan-Meier analysis is shown in Figure 3. Forty-one patients had a thrombosed occlusion of the PAA (Table 5) and were treated with a mechanical thrombectomy device. Of these, 65.9% had an acute symptomatic PAA and 34.1% had been symptomatic for 4 weeks. In none of the patients did the symptoms persist longer. Nearly two-thirds (63.4%) of these patients had additional lysis therapy. Figure 4 shows the Kaplan-Meier curve regarding TLR-free survival for the patients with an occlusion and with non- occluded PAA. Primary patency rates were 69.1%, 52.3% and 42.6% at 6, 12 and 24 months, respectively (Supplement Figure 1). Within the 6-month follow-up period, 33.6% of patients who were symptomatic preinterventionally showed improvement in RBC. After 12 months, an improvement could be observed in 39.3% of these patients. Twenty-four months after the index intervention, an improved clinical stage was documented in a total of 33.6% of the patients with preinterventional symptoms (Table 4). Changes in ABI are given in Table 4. During the follow-up period, five amputations (3.6%) were performed. (Table 4). The patients requiring a major amputation already presented CLI preinterventionally (RBC 4 n=1, RBC 5 n=1). Seven of the 134 patients died within 24 months of the intervention (Table 4). Univariate logistic regression analysis revealed age as a predictor of reintervention, and in the multivariable analysis, it was treatment with lysis (Table 6).

## 4. Discussion

This study on PAA exclusion exclusively using endoprothesis, and involving a high percentage of occluded PAAs, demonstrated: Endovascular exclusion of PAA is feasible and the limb salvage rate is promising. After 2 years, however, the primary patency is low at 42.6% and the TLR rate at this time is high at 38.8%. In the present study, the primary patency rate was lower and the TLR rate higher than in other studies [11–15]. Explanatory factors for this are the symptoms, the proportion of occluded PAA’s and the outflow. A registry study (592 interventions) demonstrated a difference in the primary patency after endovascular treatment of asymptomatic and symptomatic PAA. The primary patency rate at 12 months was 67.4% in the asymptomatic patients, 57.1% in the symptomatic group and 42.9% in patients with ALI, respectively. However, only the difference between open and endovascular repair was tested for significance in this study [11]. In a study by Golchehr et al., 70 patients with 72 PAA were treated endovascularly. The proportion of symptomatic patients was 22%, which was lower than in the present study. In addition, the proportion of patients with ALI was significantly lower (9.7%). The TLR rate was 12.5%. [12]. After endovascular treatment of 15 patients, Antonello et al. observed a primary patency rate of 86.7% at 12 months and 80% at 48 months. [13]. In another study [14], 67 patients were randomized to surgical (n=42) or endovascular therapy (n=25). Overall, 23 patients (34%) were symptomatic, and 4 patients in the endovascular treatment group had acute symptoms. The primary patency after endovascular exclusion using an endoprosthesis is reported to be 71% at 5 years. Tayfur et al. treated 63 PAA with endoprothesis and reported a primary patency of 79.3% at 46.05±25.01 months. Again, the proportion of symptomatic patients was low (38.1%). The number of patients with CLI and occluded aneurysms is not reported [15]. Recanalization of thrombosed PAAs is still widely debated and not a standard approach. The Society for Vascular Surgery clinical practice guidelines on PAA 2021 [3] recommended for thrombotic and/or embolic complications of PAA a procedure according to the severity of ischemia. The focus is on urgent revascularization. The ESVS practice guidelines for the management of ALI do not recommended stent-graft implantation as the first line treatment for patients with PAA while, at the same time, improvement of outflow by thrombolysis should be considered [16]. A study of 12 patients and completely thrombosed PAA examined outcomes after thrombolysis. In 77% of cases thrombolysis restored blood flow in the popliteal artery and outflow through one or two leg vessels. The long-term outcome was poor with a cumulative patency (no definition) of 46% at 12 months. In addition, the mortality rate was very high during the 24-month period (75%)[17]. A study investigated the outcome after thrombolysis for ALI in 51 patients with an occluded PAA [18]. At 12 months, the primary patency was 59.1%. However, mechanical thrombectomy was not performed in either studies. In contrast to other studies [12,14,15], the preinterventional outflow was inferior in this study. Unlike in another studies [13], patients with poor outflow were not excluded. In the present study, there was an average of 1.3 run-off vessels. The average number of patent tibial vessels is reported to be 2.5 and 2.35±0.72 [14,15]. Primary technical success was achieved in all patients. This is consistent with numerous other studies that have also shown high technical success rates after endovascular exclusion of PAA using endoprosthesis [1, 19,20]. This relatively low overall complication rate - also in comparison to open surgical therapy - has also been shown in other studies [21]. The most common periinterventional complication (n=8, 6.1%) was a compartment syndrome. Preinterventionalyl, all patients with periinterventional compartment syndrome had an aneurysm occlusion, six of these patients suffered ALI. All patients underwent lysis and all but one underwent additional treatment of the lower leg vessels. Teßarek observed one compartment syndrome after endovascular intervention followed by arterial lysis therapy in a group of five patients with thrombosed PAA and critical ischemia [22]. Dorweiler et al. reported compartment syndrome with subsequent fasciotomy in 16% of emergency and 3% of elective open PAAs. Emergency procedures included operations due to acute aneurysm occlusion with consecutive critical ischemia and ruptured aneurysms [23]. Jungi et al. reported about 51 patients undergoing surgical revascularization for acute thrombosed PAA. Twenty-nine% of these patients required fasciotomy due to compartment syndrome [24]. Compartment syndrome is a complication that requires special attention, especially in patients with ALI. Clinical symptoms expressed as RBC decreased significantly at all follow-up time points. Here, a comparison with other studies is difficult, because often a differentiation was only made between symptomatic or asymptomatic and the symptoms were not reported as the RBC [15,19]. The ABI improved significantly only initially after the intervention; it was not significantly changed at the other time points compared to preintervention. This can be explained because, as described, numerous restenoses occurred during the follow-up, causing the ABI to decrease.

Considering the high preinterventional rate of occluded PAAs, the low amputation rate should be mentioned even though reinterventions are often necessary for this. In the study investigating the outcome after thrombolysis of occluded PAAs, the amputation rate at one year was reported to be 27.1% [18]. A review examined the outcome after thrombolysis or thrombectomy followed by bypass surgery in patients with acutely thrombosed PAA and showed a major amputation rate within 30 days of 14.1% [25]. In the cohort of Jungi et al. a 30-day major amputation rate of 16% was reported [24]. More recent studies have shown lower amputation rates (0–1.5%) [13,14,26]. The high rate of limb salvage can most likely be explained by the simultaneous treatment of the lower leg vessels. In addition to lysis therapy, at least one lower leg vessel was treated in 71.9% of the patients with occluded PAA. Thus, in this study, 64.9% of patients had two- or three-vessel postinterventionally. Jungi et al reported a one-vessel runoff after revascularization in 57%. Only 11 % had a two patent crural runoff vessels and 26% a three-vessel runoff.

## 5. Limitations

The patient cohort was not randomly generated, therefore, the possibility of sampling bias must be considered. Differences in patient and lesion characteristics may have an impact on outcome. In addition to endoprosthesis implantation, treatment varied; a small proportion was also treated with a drug-coated balloon, which may affect the patency and amputation rate. The antiplatelet therapy was recommended for 6 months. However, numerous patients also had oral anticoagulation for various indications, which makes comparison somewhat more difficult, but corresponds to a real-world population. In conclusion, although limited by the fact that it is a retrospective study, this study can show that endovascular treatment using endoprosthesis often entails reinterventions, but is associated with a high limb salvage rate - even after recanalization and exclusion of occluded aneurysms.

## Supplementary Material

1

## Figures and Tables

**Table 1:** Baseline Characteristics

Baseline Characteristics	N=134 (%)
Age, Years	68.3±10.6
Male sex	119 (88.8)
Hypertension	107 (79.9)
Diabetes mellitus	26 (19.4)
Hyperlipidemia	105 (78.4)
Current Smoker	59 (44.0)
Former Smoker	23 (17.2)
Coronary heart disease	47 (35.1)
Cerebral vascular disease	6 (4.5)
Chronic obstructive pulmonary disease	12 (9.0)
Renal insuffciency (clearance < 60 ml/min)	40 (29.8)
Rutherford-Becker class	
0	9 (6.7)
1	22 (16.4)
2	12 (9)
3	48 (35.8)
4	32 (23.9)
5	11 (8.2)
Medication at discharge	
Dual antiplatelet therapy	73 (54.5)
Oral anticoagulation Indication: Atrial fibrillation	1 (0.7)
Oral anticoagulation and antiplatelet therapy Indication 15 Atrial fibrillation, 19 aneurysm contralateral, 11 Residual thrombus, 7 Deep vein thrombosis/pulmonary embolism, 6 Recurrent peripheral occlusions, 1 Ventricle thrombus, 1 multiple incompatibilities	60 (44.8)

**Table 2:** Lesion and Index Procedure Characteristics

Lesion and Index Procedure Characteristics	N (%)
**Lesion anatomy**	
Aneurysm diameter (cm)	2.5±0.9
P1	13 (9.7)
P2	26 (19.4)
P3	3 (2.2)
P1 and P2	32 (23.9)
P2 and P3	8 (6.0)
P1 to P3	33 (24.6)
Femoropopliteal to Popliteal 1	6 (4.5)
Femoropopliteal to Popliteal 2	8 (6.0)
Femoropopliteal to Popliteal 3	5 (3.7)
Target lesion occlusion	55 (41.0)
**Target lesion therapy**	
Plain old balloon angioplasty	61 (45.5)
Drug-coated balloon	13 (9.7)
Mechanical recanalization and thrombectomy	41 (30.6)
Catheter-directed pharmacologic thrombolysis	37 (27.6)
Lysis duration (hours)	17 [7.5]
Atherectomy	1 (0.7)
Number of endoprothesis	1–3, median 1.0
Endoprothesis Diameter (mm)	8.0±1.5
Cumulative Endoprothesis length (mm)	167.91±84.9
Lesion length (mm)	121.0±78.77
**Non-target lesion interventions**	
Inflow	38 (28.4)
Outflow	63 (47.0)
**Number of preinterventional outflow vessels**	
Zero	49 (36.6)
One	27 (20.1)
Two	30 (22.4)
Three	28 (20.9)

**Table 3:** Procedural Adverse Events

Procedural Adverse Events	22 (16.4)
**Access site complication**	6 (4.5)
**Perforation (target lesion)**	2 (1.5)
**Distal Embolization**	4 (3.0)
**Compartment syndrome**	9 (6.7)
**Complication treatment**	
- Conservative	7 (5.3)
- Endovascular	6 (4.5)
- Surgical (Fasciotomy due to compartment syndrome)	9 (6.7)

Values are n (%)

**Table 4:** Clinical and Procedural Outcomes

**Clinical and Procedural Outcomes**	
**Rutherford-Becker Class**	median, mean
Baseline (n=134)	3, 2.8±1.4
Follow-up 6 months (n=80)	1, 1.5±1.5 (p<0.001)
Follow-up 12 months (n=85)	1, 1.8±1.5 (p<0.001)
Follow-up 24 months (n=62)	1, 1.3±1.4 (p<0.001)
**Ankle-Brachial-Index**	Median, mean
Baseline (n=72)	0.9, 0.79±0.4
Post-procedure (n=117)	1.0, 0.97±0.2 (p<0.001)
Follow-up 6 months (n=65)	0.9, 0.86±0.3 (p=0.430)
Follow-up 12 months (n=65)	0.9, 0.84±0.3 (p=0.750)
Follow-up 24 months (n=51)	0.9,0.88±0.2 (p=0.041)
Target Lesion Revascularisation at 12 months	42 (31.3)
Target Lesion Revascularisation at 24 months	52 (38.8)
Endovascular reintervention	49 (94.2)
Open, surgical treatment	3 (5.8)
**Major amputation**	2 (1.5)
**Minor amputation**	3 (2.1)
**Death**	
12 months	5 (3.7)
24 months	7 (5.2)

Values are n (%) or mean ± SD

**Table 5:** Baseline Characteristics for patients with occluded PAA and without occluded PAA

Baseline Characteristics	Perfused PAA	Occluded PAA	p-value
Age, Years	68.7±10.8	67.7±10.4	0.596
Male sex	67 (84.8)	52 (94.5)	0.098
Hypertension	67 (84.8)	40 (72.7)	0.124
Diabetes mellitus	18 (22.8)	8 (14.5)	0.273
Hyperlipidemia	66 (83.5)	39 (70.9)	0.092
Smoker	44 (55.7)	38 (69.1)	0.150
Coronary heart disease	34 (43.0)	13 (23.6)	0.027
Cerebral vascular disease	5 (6.3)	1 (1.8)	0.400
Chronic obstructive pulmonary disease	7 (8.9)	5 (9.1)	1.000
Renal insuffciency (clearance < 60 ml/min)	24 (30.4)	16 (29.1)	1.000
Rutherford-Becker class			
1	22 (27.8)	0	<0.001
2	10 (12.7)	2 (3.6)	0.122
3	24 (30.4)	24 (43.6)	0.143
4	9 (11.4)	23 (41.8)	<0.001
5	5 (6.3)	6 (10.9)	0.358

**Table 6:** Predictors of Target Lesion Revascularization

Univariate Analysis				Multivariable Analysis		
	OR	95%-CI	p-Value	OR	95%-CI	p-Value
Age (per year)	0.960	0.917–0.994	0.022	0.943	0.887–1.002	0.058
Sex	0.945	0.316–2.831	0.920	2.139	0.360–12.693	0.403
Hypertension	0.511	0.218–1.199	0.123	0.717	0.204–2.514	0.603
Hyperlipidemia	1.0481	0.450–2.444	0.913	1.974	0.516–7.552	0.321
Diabetes mellitus	0.684	0.272–1.724	0.421	0.771	0.196–3.032	0.710
Smoker	2.081	0.946–4.580	0.069	1.762	0.437–7.097	0.302
Coronary heart disease	0.842	0.404–1.753	0.646	2.118	0.510–8.803	0.302
Stroke	0.302	0.034–2.661	0.281	0.374	0.030–4.700	0.446
Renal insufficiency	0.764	0.347–1.683	0.504	0.783	0.226–2.704	0.698
						
Critical limb ischaemia	1.848	0.883–3.867	0.103	2.042	0.531–7.861	0.299
Target lesion occlusion	1.410	0.697–2.853	0.339	1.286	0.214–7.719	0.784
Inflow treatment	0.890	0.410–1.934	0.769	0.581	0.157–2.145	0.415
Outflow treatment	0.833	0.414–1.673	0.607	2.265	0.473–10.842	0.306
BTK preinterventional	0.969	0.718–1.307	0.836	0.731	0.107–4.995	0.750
BTK postinterventional	1.166	0.750–1.811	0.495	0.834	0.184–3.790	0.814
Viabahn Diameter	0.974	0.776–1.224	0.822	1.000	1.000	0.984
Cumulative Viabahn Length	1.002	0.998–1.006	0.279	1.000	0.992–1.009	0.938
Mechanical recanalization and thrombectomy	2.100	0.993–4.441	0.052	2.824	0.447–17.826	0.269
Lysis	1.106	0.510–2.397	0.799	0.185	0.037–0.993	0.041

BTK – below the knee, CI – confidence interval, OR – odds ratio

Statistical significance was set as p<0.05.
