# Supplementary figures and images for: Early Results after Exclusion of Popliteal Aneurysms with an Endoprosthesis
Source: Cardiol Cardiovasc Med. Author manuscript; Available in PMC 2023 Feb 10. (PMC9912976; doi:10.26502/fccm.92920298)

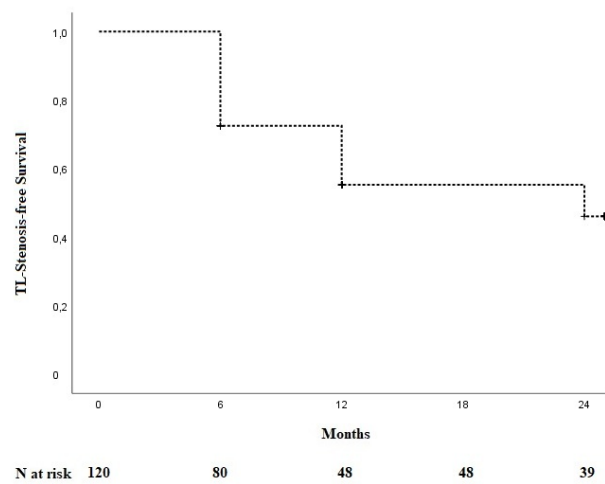

**Figure Supplement 1:** Kaplan-Meier curve for TL-stenosis-free survival.

TL-target lesion

Supplement: 1 [file NIHMS1865647-supplement-1.pdf]
